# Fast recruiting clinical trials – a Utopian dream or logistical nightmare?

**DOI:** 10.1038/sj.bjc.6602574

**Published:** 2005-04-19

**Authors:** L Johnson, P Ellis, J M Bliss

**Affiliations:** 1 ICR-CTSU, Section of Clinical Trials, Brookes Lawley Building, Institute of Cancer Research, Cotswold Road, Sutton, Surrey SM2 5NG, UK; 2 Department of Medical Oncology, Guy's Hospital, St Thomas Street, London SE1 9RT, UK

**Keywords:** large phase III clinical trials, fast recruitment

## Abstract

Randomised clinical trials that exceed anticipated recruitment rates will by definition have the necessary precision to answer the research question within the expected time, thus ensuring the timely release of data that will inform future clinical practice. In addition, the national or international momentum generated brings with it a collective sense of achievement. Such trials, however, may also identify logistical and scientific problems that researchers should be aware of and for which provision needs to be made. The logistical problems relate to the rapid identification of the extra resources required to allow continued excellence in day-to-day management and monitoring of trial governance (both in participating centres and in coordinating trials units). The scientific/clinical problems include managing issues such as unexpected toxicities and suboptimal compliance, and the lack of time available in a rapidly recruiting trial to address them. A related issue concerns the lack of time available to initiate substudies (e.g. biological substudies), the relevance of which may only become apparent as the trial progresses. Many of these challenges were highlighted by recent experience with the Cancer Research UK Taxotere as Adjuvant Chemotherapy trial.

Historically, in the UK debate concerning clinical trial accrual has centred on under recruitment into national randomised trials and how we might rectify this. More recently the successful completion of the Cancer Research UK Taxotere as Adjuvant Chemotherapy (TACT) trial in early breast cancer has raised a new question. Are there problems associated with large-scale trials that recruit too quickly?

The TACT trial involved the coming together of the UK breast cancer clinical trial community on a scale not previously seen before. Five clinical trials units, 104 centres (including one Belgian centre) and 220 clinicians collaborated together to address the question of whether sequential Taxotere (docetaxel) following FEC chemotherapy was superior to standard anthracycline chemotherapy of similar duration (Barrett-Lee *et al*, 2002) (Figure 1). As a result, 4162 patients were randomised into this trial in just 28 months. While many positives can be taken from the successful completion of this trial, the scale and rapidity of recruitment identified a number of logistical and clinical trial management problems. This paper discusses these and seeks to identify solutions that may enhance the management and governance of future trials.

## THE PROBLEMS OF FAST RECRUITING TRIALS

### Logistical problems

If a large number of centres desire initiation at one time, this can be overwhelming for trials units and lead to frustration at centres and trials units alike. Delays are very likely to occur in ensuring the proper process of site initiation is achieved.

If patients are entered more rapidly than was anticipated, trials units can be overwhelmed by the rapid arrival of data. Subsequent delays in processing case report forms (CRFs), chasing missing data, and identifying and resolving data queries mean any emerging problems contained within the data, for example, treatment toxicity and/or compliance issues, are not seen and acted upon quickly. Similarly for participating centres, if a centre's resource focus is dedicated to recruiting patients, it could be at the expense of completing and returning CRFs promptly.

### Scientific implications

The scientific implications are a consequence of speeding up timelines for a trial. Many concern general issues of good trial management practice; however, the implications are greater in a fast recruiting trial due to the number of patients entered before a problem can be identified and remedial action taken.

Treatment toxicity manifests itself at a time directly related to treatment duration, for example, it can take several months for acute toxicity associated with chemotherapy and/or radiotherapy to manifest itself, and several years for any long-term sequelae of treatment to arise. Toxicity issues may relate to an unexpected type of toxicity or to an unexpectedly high rate of an anticipated adverse event. If expected serious adverse events (SAEs) are exempt from expedited reporting to the trial coordinators, as is common in many trials these days, then such problems will only become apparent when CRFs are finally received and processed. If the recruitment rate is very high, however, a large number of patients will have been recruited and more importantly treated, in the intervening period. Similar arguments apply to issues related to treatment compliance; recruitment continues while the existence and cause of any problem is uncovered.

Conducting a quality of life (QL) substudy within the main trial illustrates a number of these issues. The exact sample size required for a QL study is often not calculated with certainty at the start of a trial and is therefore often presented as a range. The proportions of patients completing both QL follow-up assessments and trial treatment determines the actual sample size once the trial is underway. If the estimated upper limit of recruitment is reached before these determining factors can be assessed, researchers must then either increase the sample size to account for hypothetical noncompliance with either the treatment or the QL assessments, or stop recruitment to the QL study and hope that any noncompliance is within the bounds used to calculate the upper limit of the original estimate.

Very rapid accrual to the main trial can also mean that late initiation of correlative science substudies requiring fresh material (e.g. blood) could result in the collection of fewer samples. For example, patients entered earlier in the trial may be followed up at an alternative hospital and gaining additional consent may be less feasible. Furthermore, if such studies are activated, with requests being made to all patients entered into the trial to date, laboratories processing samples can themselves experience unmanageable workload issues.

The typical efficacy end point of a randomised phase III trial in nonmetastatic cancer is 5-year disease-free survival, and typically the statistical analysis plan will define the observed number of events expected to be observed before the trial has sufficient power to detect reliably clinically important differences between treatment groups. This number will have been devised according to planned recruitment and event rates and will be expected to be an average of early and late occurring events. If recruitment is swift, the required number of events can be observed, with weighting towards early events, before the duration of clinical follow-up is mature enough to have sufficient weight to alter clinical practice, irrespective of the statistical significance of the observed treatment effect. This is a particular problem where the treatments under investigation involve prolonged administration (e.g. endocrine therapy in breast cancer). In metastatic disease trials, where the end point is progression-free survival, the consequences of fast recruitment are unlikely to be problematic since, the time taken to observe progression is often considerably shorter than the time required to observe or exclude relapse in the adjuvant setting. Hence, fast recruitment will simply lead to a swifter conclusion of the trial.

## THE TACT TRIAL EXPERIENCE

**Figure 1:**
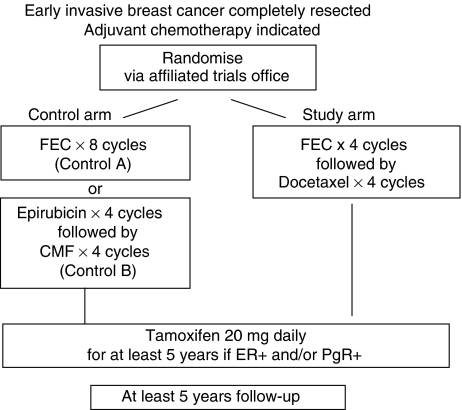
TACT trial design.

**Figure 2:**
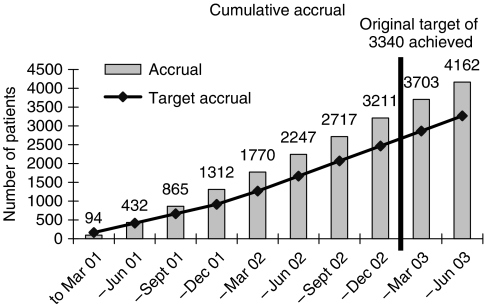
Target and actual accrual into the Taxotere as Adjuvant Chemotherapy (TACT) Trial.

The TACT trial was a phase III randomised trial in early breast cancer comparing different chemotherapy schedules (Figure 1). The treatments under investigation were well established in metastatic disease and as with all chemotherapies were expected to be associated with a degree of toxicity. The TACT trial aimed to recruit 3340 patients in 3 years from 80 centres, with anticipated monthly accrual peaking at 100 patients. A mid-trial protocol amendment increased the target sample size to 4000. Actual recruitment was 4162 patients from 104 centres over 28 months, peaking at over 170 patients a month (Figure 2).

### Logistical problems

The Clinical Trials & Statistics Unit at the Institute of Cancer Research (ICR-CTSU) acted as the coordinating centre for TACT (‘hub’), with four other UK trials offices acting as ‘spokes’ for randomisation and data management on behalf of local and regional research affiliations. Data arrived at all trials offices at a faster rate than it could be processed. Consequently, regular analyses of safety data on frozen data sets did not include the most recent data, or the data accumulating while the analyses were conducted. The Data Monitoring and Ethics Committee (DMEC) could therefore only ever base recommendations on relatively historical data.

### Scientific implications

Arthralgia and myalgia during treatment with Taxotere emerged as a more frequent and severe toxicity than had been anticipated. Taxotere was administered during the second phase of chemotherapy treatment, which began 12 weeks after randomisation. The trial coordinator was alerted to the emerging problem by nurses telephoning ICR-CTSU, but more than 1000 patients were randomised before data from the first group of patients randomised were available for review. The Chief Investigators immediately informed all participating clinicians, and the patient information sheet (PIS) was amended to highlight this toxicity. However, it was several weeks before the revised PIS was approved by the Multi Research Ethics Committee (MREC), with further delays in adopting it in some centres caused by their local ethics procedures. As a result, many more patients were recruited before the remedial action taken was fully implemented, with the duration of treatment and logistical problems of data handling both contributing to a further 6 months elapsing before the full effects of the remedial action could be assessed.

The total duration of chemotherapy treatment in TACT was 26 weeks, the time taken for sufficient research nurses to alert the trial coordinator to an unexpected treatment compliance profile, which prompted a formal review of the compliance data. This revealed a potential for suboptimal compliance to be a problem within centres using the FEC control arm. It seemed likely that it was caused by the way the trial and/or the treatments were being perceived. Trial patients within those centres were often treated alongside non-trial patients receiving a six-cycle regimen, and in that setting commitment to completing eight cycles was challenged for both the control and experimental arms. The Chief Investigators alerted all participating clinicians to the potential problem, and the PIS was revised, but fully implementing it was subject to the delays similar to those outlined above. Wording of the PIS involved a sensitive balance of perceptions. Whilst there was consensus that patients must be told they may withdraw from a trial at any time without giving a reason, there was disagreement about telling patients that trial results may be compromised if patients withdrew for reasons other than treatment intolerability. As a result of the time lag between randomising patients and compliance data emerging, careful monitoring of compliance was always historic. Had the problem persisted, the high recruitment rate meant that accrual would have ended before further action could be taken.

The recruitment plan for the QL Study was 500-700 patients recruited over 2 years from 20 to 25 centres. The follow-up assessments continued for 2 years. However, 829 patients were entered in 1 year from 41 centres. Recruitment was suspended to allow an evaluation of compliance with follow-up booklets and treatment, which would determine whether to reopen the QL Study. Full compliance was still impossible to evaluate 6 months later, and there were, in any case, no resources available to reactivate the QL Study. Fortunately, later data confirmed that compliance with booklets was sufficiently high.

For the TACT correlative science studies, the first requests for paraffin blocks were delayed by 5 months because of trials office workload. Although there were no long-term consequences, reference laboratories were overwhelmed by the rapid arrival of paraffin blocks, with consequent delays in returning the first blocks back to pathology laboratories. The collection of blood samples for a further biological study was delayed until 5 months before recruitment ended. For many centres, earlier patients were being followed up elsewhere, making additional consent difficult to obtain. This has resulted in the number collected being approximately 40% below planned levels.

Plans for other add-on studies looking at cardiac toxicity, ovarian function and bone density were halted when it became clear that the scale and speed of recruitment meant these studies would not be completed before the main trial had completed accrual. While failure to conduct these add-on studies by no means compromised the overall value of the main trial, a window of opportunity was missed, and questions posed by these studies remain unanswered.

## THE POSSIBLE SOLUTIONS

**Figure 3:**
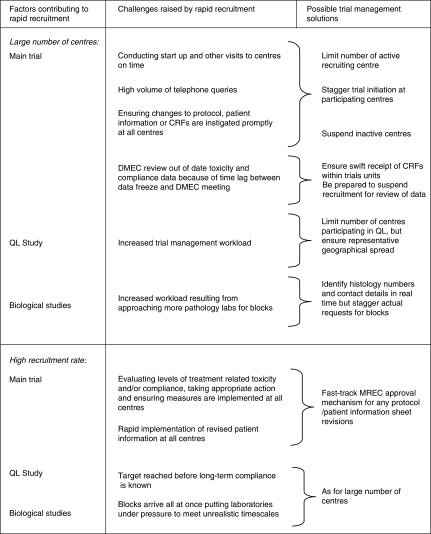
Trial management solutions to challenges raised by rapid recruitment.

The TACT trial has shown the power of collaboration. It has also identified problems that if solved can enhance the conduct of future large randomised clinical trials (Figure 3).

It would seem sensible to optimise, not maximise, both the accrual and the number of participating centres. This could be achieved by staggering initiation of trial sites and targeting sites with known experience or expertise. Careful selection will ensure all sites enter patients who would not otherwise be offered trial entry, and not draw recruitment away from existing centres with more expertise. It will also ensure adequate resources to support the trial in terms of staff experienced in gaining informed consent and completing CRFs, pharmacy support and R&D infrastructure.

Most trials are resourced via project grants awarded for each specific trial and the level of resource requested based on anticipated recruitment rates. While extra funding, from NCRN, for UK trial units running fast recruiting trials has been very welcome, it has been responsive. Hence, it is still necessary to show that a problem exists before further funding is awarded, with the consequence that further time is needed to recruit and train staff. If fast recruiting trials are to become more common, then additional core funded experienced trial coordinators working flexibly across trials, who are able to respond quickly to unexpectedly high levels of incoming data or requirements for centre visits could overcome this issue.

Better toxicity and adverse event reporting can be achieved by carefully considering what information is not urgently required and can be requested on the CRFs, and what information requires the expedited reporting mechanism used for SAEs. Only suspected unexpected serious adverse reactions (SUSARs) require expedited reporting to the MHRA. Expedited reporting to trial coordinators (trials unit and chief investigator) of some or all expected SAEs could serve as a useful early warning mechanism for unexpected rates of known SAEs. This will allow real-time processing of SAEs (after clinical review) to monitor incidence (utilising formal sequential analysis techniques), and interim safety analysis after a minimum of a given number of patients or period of time whichever occurs first.

Monitoring treatment compliance is dependent on the timely collection of information at each stage of treatment and is helped if only essential information is collected and the overcollection of irrelevant detail avoided. Well-designed CRFs will capture essential details at each stage of treatment (e.g. chemotherapy cycle) in a timely fashion, thus allowing sequential monitoring of treatment compliance for each treatment arm and each centre per calendar period (to isolate a learning effect). However, real-time analysis can only be carried out if participating centres meet CRF return targets.

By identifying funding for substudies in parallel with the main trial and ensuring that details of all substudies are included in the final protocol and in the PIS and consent forms, they can be launched at the outset of the trial or very shortly after. Specimen processing procedures need to be negotiated, which are manageable and achieve realistic timelines so that central laboratories and participating centres are prepared for the workload of biological substudies. Some centres may not be able to accommodate extra workload associated with certain substudies (e.g. QL), and this should be considered at site selection but should not preclude a centre from the trial, providing participation in that substudy is optional. The complexities of running a portfolio of substudies need to be recognised at the outset.

## CONCLUSIONS

Process and governance require regular and timely input from those overseeing a trial, that is, the trials unit, Chief Investigator and substudy coordinators, the DMEC, the Trial Management Group and Trial Steering Committee. Flexibility in trial-related resources within trials units allows real-time data processing and analysis of accumulating data. Rapid submission of amendments to COREC and the MHRA, and efficient communication channels with participating centres are both crucial.

While the experience described here relates to the conduct of a UK trial, many of the issues raised are relevant for international trials with complex logistics and remedial strategies. As international collaboration increases, it is likely that the so-called ‘over-recruiting’ trials will become more common. Requirements of such trials need to be anticipated, dynamic management strategies and flexible resourcing identified, thus allowing proper management of such trials while avoiding excess resource allocation.

Fast recruiting trials bring benefits and instil huge enthusiasm; they inspire collaboration within both trials units and participating centres and ensure that large numbers of patients are entered into clinical trials. This can result in the speedy conclusion of a trial that can inform clinical practice. However, the need to ensure adequate resources and capacity are available to manage fast recruiting trials should not be underestimated Researchers must be prepared to act quickly, if required, to amend the protocol or PIS. They also need to ensure that substudies are ready from the outset. This is simply good practice which if overlooked in a slow recruiting trial results in frustration and inconvenience; for a fast recruiting trial it could spell disaster.

Fast recruiting clinical trials are not a dream; they are now a reality. They are not however Utopian, although many of the potential pitfalls can be avoided if all involved in running a trial plan ahead and communicate effectively. Adequate resources will not resolve scientific consequences that are a direct result of fast recruitment, but logistical nightmares can be avoided if the consequences of over-recruitment are foreseen, and a plan of action put in place in future clinical trials.
